# Optimising the conservation of genetic diversity of the last remaining population of a critically endangered shrub

**DOI:** 10.1093/aobpla/plab005

**Published:** 2021-01-09

**Authors:** William J W Thomas, Janet M Anthony, Mark P Dobrowolski, Siegfried L Krauss

**Affiliations:** 1 School of Biological Sciences, The University of Western Australia, Crawley, WA, Australia; 2 Kings Park Science, Department of Biodiversity, Conservation and Attractions, Kings Park, WA, Australia; 3 Iluka Resources Ltd, Perth, WA, Australia; 4 Harry Butler Institute, Murdoch University, Murdoch, WA, Australia

**Keywords:** Mating system, microsatellites, paternity, spatial genetic structure, species recovery, *Styphelia longissima*, translocation

## Abstract

An understanding of genetic diversity and the population genetic processes that impact future population viability is vital for the management and recovery of declining populations of threatened species. *Styphelia longissima* (Ericaceae) is a critically endangered shrub, restricted to a single fragmented population near Eneabba, 250 km north of Perth, Western Australia. For this population, we sought to characterize population genetic variation and its spatial structure, and aspects of the mating portfolio, from which strategies that optimize the conservation of this diversity are identified. A comprehensive survey was carried out and 220 adults, and 106 seedlings from 14 maternal plants, were genotyped using 13 microsatellite markers. Levels of genetic variation and its spatial structure were assessed, and mating system parameters were estimated. Paternity was assigned to the offspring of a subsection of plants, which allowed for the calculation of realized pollen dispersal. Allelic richness and levels of expected heterozygosity were higher than predicted for a small isolated population. Spatial autocorrelation analysis identified fine-scale genetic structure at a scale of 20 m, but no genetic structure was found at larger scales. Mean outcrossing rate (*t*_m_ = 0.66) reflects self-compatibility and a mixed-mating system. Multiple paternity was low, where 61 % of maternal siblings shared the same sire. Realized pollen dispersal was highly restricted, with 95 % of outcrossing events occurring at 7 m or less, and a mean pollen dispersal distance of 3.8 m. Nearest-neighbour matings were common (55 % of all outcross events), and 97 % of mating events were between the three nearest-neighbours. This study has provided critical baseline data on genetic diversity, mating system and pollen dispersal for future monitoring of *S. longissima*. Broadly applicable conservation strategies such as implementing a genetic monitoring plan, diluting spatial genetic structure in the natural population, genetically optimizing *ex situ* collections and incorporating genetic knowledge into translocations will help to manage the future erosion of the high genetic variation detected.

## Teaser

Styphelia longissima (Ericaceae) is a critically endangered shrub restricted to a single fragmented population near Eneabba, 250 km north of Perth, Western Australia. Here, 13 microsatellite markers were used to genotype 220 adults and 106 seedlings to assess genetic diversity and structure, mating system parameters and pollen dispersal. The only population of S. longissima was found to be characterised by surprisingly high levels of genetic diversity, with small groups of related individuals within which the vast majority of mating occurs. This study has provided critical baseline data for future monitoring and to inform conservation strategies.

## Introduction

Genetic diversity is widely acknowledged as a critical, yet under-utilized, consideration for the conservation management of threatened species (Allendorf *et al*. 2013; Taylor *et al*. 2017; Laikre *et al*. 2020). Conservation genetics research seeks to characterize current levels and patterns of genetic diversity within and among populations and species, with an initial aim of identifying units of conservation and prioritizing populations and species for more urgent intervention (Coates *et al*. 2018). Once units of conservation are identified, a major objective of conservation genetics is to address the genetic factors that pose a threat to the survival of threatened species and populations (Frankham *et al*. 2017; Kramer and Havens 2009). For studies such as these, the focus is on the processes that impact future genetic diversity, and the link between genetic diversity and fitness of populations. Small isolated populations that have had their distribution recently reduced and fragmented are particularly prone to losses in genetic diversity associated with increased inbreeding, genetic drift and reduced gene flow (Ellstrand and Elam 1993; Frankham *et al*. 2002,^ 2017^). For species that are represented by a single population, such losses can be permanent and, therefore, extremely detrimental (Edwards *et al*. 2014). Thus, understanding the process of mating and the factors influencing mating is important to determine its impact on reproduction, future genetic diversity, population viability, ecological resilience and ultimately evolutionary potential (Coates *et al*. 2007; Yates *et al*. 2007; Frankham *et al*. 2017).

Plant mating systems are defined as the genetic relatedness and pairings between plants in a population, not to be confused with plant breeding systems, which more commonly refers to the anatomical and physiological aspects of a plant’s reproductive system (Neal and Anderson 2005). Mating systems are influenced by several factors, including breeding system parameters (e.g. self-compatibility, floral structure), the ecological attributes of a population (e.g. size, shape, landscape position, habitat quality) and the type of pollinator (e.g. vertebrate or invertebrate) (Coates *et al*. 2007; Llorens *et al*. 2012; Barrett and Harder 2017). Consequently, mating system parameters can vary enormously within and among individuals and populations of a species (Whitehead *et al*. 2018). Identifying and understanding the drivers and consequences of spatial and temporal changes in mating patterns is vital to the conservation of threatened species, and should be considered when devising a conservation strategy (Pierson *et al*. 2016). This is particularly true for species which have undergone substantial environmental disturbance. For example, outcrossing rates are typically lower in disturbed plant populations than undisturbed ones (Eckert *et al*. 2010). A reduction in outcrossing rate can be brought about by the impacts of habitat fragmentation and population size reduction on pollinators and pollination quantity (number of pollinators, visits and pollination events) and quality (number of diverse pollen grains belonging to the same or another species)—an example of the Allee effect (Lamont *et al*. 1993). As a result, Allee effects on pollinators can reduce the quantity (seed set) and/or genetic quality (increased inbreeding) of seed, potentially impacting population viability, resilience to environmental change and extinction risk.

**Figure 1.**
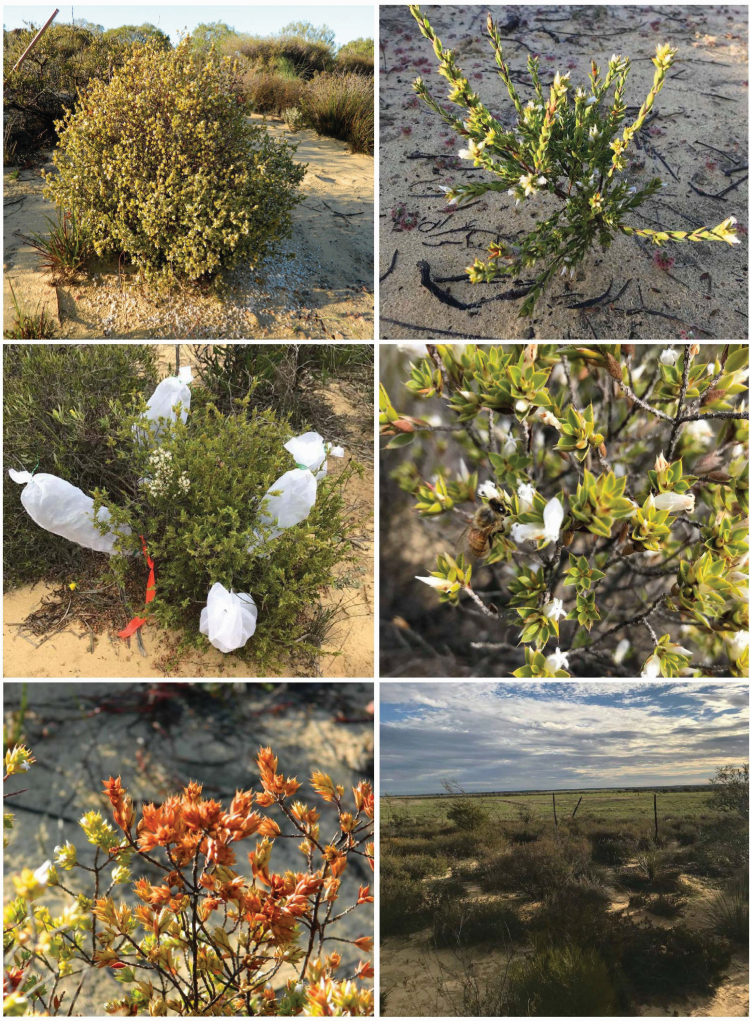
*Styphelia longissima* growing in an isolated remnant of native kwongan heath near Eneabba, Western Australia. Photos include a large adult (top left), a juvenile (top right), a plant bagged for seed collection (centre left), a European honey bee (*Apis mellifera*) foraging for pollen (centre right), an unhealthy plant (bottom left) and vegetation within the site surrounded by cleared land (bottom right).

*Styphelia longissima* (Ericaceae) is a recently described and clearly distinct species found near Eneabba, Western Australia (Fig.1; Hislop and Puente-Lelièvre 2017). It is described as a spindly to dense perennial shrub that grows on yellow sand and has white flowers that appear between May and July each year. *Styphelia longissima* occurs in kwongan heathland, a floristically rich and diverse community of some 5600 plant species on low nutrient sandy soils experiencing a Mediterranean type climate (Byrne *et al*. 2014). *Styphelia longissima* is restricted to a single population which was discovered in 2003, located in an isolated 13.5 ha remnant patch of native vegetation situated among agricultural fields, with the nearest remnant vegetation being 1 km away (Woodman Environmental Consulting Pty Ltd 2003, 2006, 2008). *Styphelia longissima* is listed as Critically Endangered under the Western Australian Biodiversity Conservation Act 2016.

The future of this taxon *in situ* currently relies upon the viability of this single remaining population of adult plants, and conservation action is urgently needed. A current lack of understanding of many fundamental aspects of the biology of *S. longissima*, and indeed many of its congeners, impedes active conservation. Currently, there are no data relating to its genetic diversity, mating system, breeding system, pollination biology, seed dispersal, soil seed bank persistence, seed dormancy, germination ecology, longevity or fecundity. Based on limited pollinator observations on the closely related genus *Leucopogon*, it is likely that *S. longissima* is insect pollinated, as are most species in the Ericaceae (Keighery 1996; Johnson 2013). Seed dispersal is primarily by gravity, and the presence of an elaiosome promotes secondary dispersal by ants, probably at a scale of metres (Harris 2013; Pascov *et al*. 2015).

The present study aims to assess genetic diversity and its spatial structure in *S. longissima* and to characterize its mating system, breeding system and pollen dispersal. This study is also the first to examine population genetic diversity, spatial genetic structure (SGS), mating system and pollen dispersal for any member of the Australian Ericaceae (subfamily Epacridoideae), a large group containing ca 420 species (Crayn *et al*. 2014). It is also one of the first to shed light on the ecological genetics of a threatened plant species of the diverse kwongan (Zawko *et al*. 2001; Byrne *et al*. 2014).

Specifically, the following questions are addressed: What is the current population size and demography? What are the levels of genetic variation in the population? How is it spatially structured? Is *S. longissima* self-compatible, and if so, what proportion of mating is selfing vs outcrossing? How does mating frequency change with geographic distance between mates? It is predicted that pollination by insects will result in restricted pollen dispersal, considerable near-neighbour mating and low rates of outcrossing if self-compatible, thus causing high levels of inbreeding and fine scale SGS. In this way, the current study establishes a baseline of understanding of population genetic pattern and process to underpin future genetic monitoring and assessment of conservation and translocation efforts, and to identify strategies for the management of genetic diversity for conservation.

## Methods

### Plant mapping and sample collection

**Figure 2.**
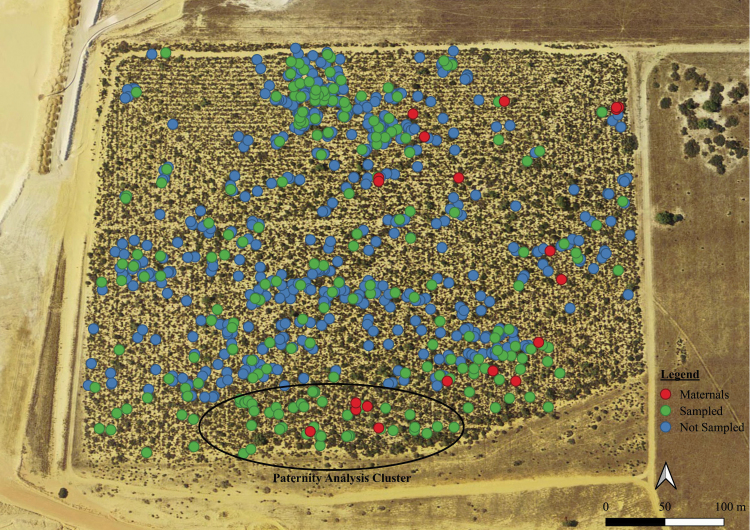
Distribution of *Styphelia longissima* in an isolated block of remnant vegetation near Eneabba, Western Australia. Red dots are maternal plants sampled for seed, green dots are plants sampled for DNA and blue dots are plants which were not sampled. Plants that were used in paternity analysis are shown.

An exhaustive survey of all known plants was carried out and the GPS coordinates of each individual were recorded using a Trimble Geo7X GeoExplorer differential GPS (Fig. 2). The relative health of each plant was assessed. For example, vigorous green growth was deemed healthy and significant browning of the leaves was deemed stressed. Each plant was also recorded as an adult or juvenile based on size. A section of healthy leaves was sampled from an arbitrarily selected subsample of 220 plants which covered the entire species distribution. Samples were stored in plastic zip-lock bags and stored at 4ºC. Nylon mesh bags were placed over five branches on each of 20 arbitrarily selected individuals and an average of 239 fruits were collected per plant. Fruits were stored in envelopes at room temperature.

### Seed preparation

To determine the viability of the fruit, which can contain either one or two seeds, X-ray images were taken using a Faxitron^®^ MX-20 digital X-ray (Faxitron Bioptics, USA) to check for underdeveloped seeds. On average, 20 % of the fruit from each maternal plant were non-viable and were discarded. Seeds were extracted, sterilized and plated following Harris (2013). Seeds were incubated at 15ºC under a 12-h day/night cycle and observed daily for germination.

### DNA extraction, PCR amplification and microsatellite screening

Genomic DNA was extracted from 220 adult leaf tissue samples and 106 whole seedlings (hereafter referred to as the adult and seedling cohorts), following the Carlson extraction method (Carlson *et al*. 1991) with modifications as per Anthony *et al*. (2017). Thirteen primer pairs designed for microsatellite amplification in *S. longissima* were used in four multiplex primer mixes and reactions were carried out, as per Anthony *et al*. (2017). PCR products were visualized by electrophoresis on an automated 3500 Genetic Analyzer (Applied Biosystems) by adding 1 µL of amplified PCR product to 8.9 µL Hi-Di Formamide and 0.1 µL 500 LIZ size standard (Applied Biosystems). Alleles were scored manually using Geneious V7.1.4 (Kearse *et al*. 2012). To ensure PCR was carried out correctly and there was no contamination, a negative and positive control were added to every PCR run. A list of alleles at each locus was generated using GenAlEx V6.51b2 (Peakall and Smouse 2012) to check for scoring error. Micro-Checker (Van Oosterhout *et al*. 2004) was used to test for the presence of null alleles and allelic drop-outs. Replicate runs were carried out to ensure microsatellite reproducibility. Family arrays were screened to check for Mendelian inheritance of the markers. Final genotypes were double-checked manually.

### Genetic diversity

The following parameters were measured per locus using GenAlEx for both the adult and seedling cohorts: sample size (*N*), number of alleles (*N*_a_), effective number of alleles (*N*_e_), observed heterozygosity (*H*_O_), expected heterozygosity (*H*_E_) and fixation index (*F*). A Wilcoxon’s rank sum test was used to test for significant differences in *H*_*O*_, *H*_*E*_ and *F* between adults and seedlings in R (R Core Team 2017). Allelic richness, corrected for sample size, was calculated for both cohorts in FSTAT V2.9.4 (Goudet 1995).

### Genetic differentiation and spatial structure

The number of distinct genetic clusters (K) within the population was assessed in the adult cohort using the program STRUCTURE V2.3.4 (Pritchard *et al*. 2000). A model that specified no prior groupings was selected and different K values (1–5) were tested. A burn-in period of 100 000 and 10^5^ Markov-Chain Monte Carlo randomizations were used and 10 independent iterations for every K value were performed. A web-based Python program (STRUCTURE Harvester, Earl and vonHoldt 2012) selected the K value that best fit the data, using the Evanno method (Evanno *et al*. 2005). A Principal Coordinates Analysis of genetic distance was generated in GenAlEx to visualize clustering.

Pair-wise matrices of genetic and geographic distance between all adult individuals were generated and a Mantel test was used to test their relationship. In addition, spatial autocorrelation analysis (SAA) was used to assess the genetic relatedness of adult individuals as a function of geographic distance. Evenly spaced distance classes of 5 m were chosen because it was the smallest distance class that maintained >30 individuals in all classes. A correlation coefficient (*r*) was calculated for all pairs of individuals within each distance class. Confidence limits (95 %) about (i) the null hypothesis of no spatial structure and (ii) *r,* were generated by 1000 bootstraps. Distance matrices, Mantel test and SAA were generated in GenAlEx V6.51b2 (Peakall and Smouse 2012).

### Mating system

Although 900 seeds were prepared, difficulties in processing and preparing seed for germination meant only 106 germinated and were genotyped. Hence, the number of seedlings in each family varied considerably. In total, the seedling cohort comprised 14 families containing between 2 and 20 seedlings, with an average of eight and a median of five seedlings per family. Parental inbreeding coefficient (*F*_m_), multilocus outcrossing rate (*t*_m_), singlelocus outcrossing rate (*t*_s_), bi-parental inbreeding rate (*t*_m_ - *t*_s_), correlation of paternity (*r*_p_) and correlation of outcrossing among loci (*r*_tl_) were estimated in the seedling cohort using MLTR (Ritland 2002). Standard errors were based on 1000 bootstraps. All measures were calculated at the population level. To assess the impact of having many small families when estimating mating system parameters, a subset of the seedling cohort was also analysed. This subset included five families; those which contained more than eight seedlings (recommended minimum family size).

### Paternity assignment and pollen dispersal

For paternity analysis, 51 adult plants which surrounded five closely grouped maternal plants (Fig. 2) were genotyped and were included as potential sires. Of the 106 seedlings that made up the seedling cohort, 37 were the offspring of the five maternal plants, and were subsequently used for paternity analysis. The most likely sire was assigned to the 37 seedlings through categorical allocation by CERVUS V3.0.7 (Kalinowski *et al*. 2007) on the basis of logarithm of odds (LOD) scores. In parentage analysis, a LOD score is an estimate of the likelihood that a candidate parent is the true parent (Meagher 1986). A strict confidence level was set at 95 % and a relaxed confidence level was set at 80 %. For any allocation resulting in a confidence level below 80 %, the most likely sire was assigned.

From paternity assignment, an outcrossing rate was calculated based on the proportion of seedlings that were assigned non-maternal plants as the most likely sire. Measures of realized pollen dispersal were generated by calculating the distance between the maternal plant and the assigned sire. The total number of sires and number of outcross sires were also calculated. The number of mating events, including selfing, was plotted against distance. To assess the extent of near-neighbour mating, a ranked distance distribution and a realized pollen distribution curve were generated. All plant pairs were ranked from 1 to 220 where 1 is the closest neighbour and 220 is the most distant neighbour. Both distributions were tested for kurtosis using the kurtosis function in R (R Core Team 2017).

## Results

### Population size and demography

A total of 947 individuals were found and mapped (Fig. 2). At the time of monitoring (June 2018), the majority of plants (94 %) were healthy and a minority (6 %) showed signs of stress. Only one dead individual was found. Adults comprised 88 % of plants and 12 % were juvenile. Individuals in full flower made up 98 % of all plants; only 1 % were budding and 1 % showed no signs of flowering. Plant height ranged 6–126 cm with the majority (75 %) of individuals 20–40 cm.

### Genetic diversity

Replicate samples of 50 adults and 20 seedlings (of the total 220 adult plants and 106 seedlings sampled) were genotyped twice and were scored identically. All families used in analyses showed Mendelian inheritance patterns. There was no evidence for allelic dropouts, but there was evidence that null alleles might be present at all but one locus. In the adult cohort, the number of alleles per locus ranged from 6 to 27 (Table 1), with a total of 147 different alleles identified. The mean allelic diversity in the seedling cohort was lower than in the adult cohort (Table 1). A wide range of observed and expected heterozygosity was seen (*H*_O_ = 0.20 – 0.85, *H*_E_ = 0.52 – 0.92). There was a heterozygote deficit at 12 of 13 loci. Heterozygosity was lower and *F* was higher in the seedling cohort than in the adult cohort. However, all three measures did not significantly differ from the adult cohort (Wilcoxon’s rank sum test; *P* = 0.336 (*H*_O_), 0.879 (*H*_E_), 0.336 (*F*)). Number of alleles was significantly higher in the adult cohort compared to the seedling cohort (Wilcoxon’s rank sum test; *P* = 0.025). Average allelic richness per locus was 10.08 for the adult cohort and 7.49 for the seedling cohort.

**Table 1.** Genetic variation across 13 microsatellite loci for *Styphelia longissima* (Anthony *et al.* 2017) in the adult cohort (*n* = 220) and the progeny (*n* = 106). Measures include sample size (*N*), number of different alleles (*N*_a_), number of effective alleles (*N*_e_), observed heterozygosity (*H*_O_), expected heterozygosity (*H*_E_) and fixation index (*F*). Standard error is shown in parentheses.

Locus	*N*	*N*_*a*_	*N*_*e*_	*H*_O_	*H*_E_	*F*
Sl53	212	6	2.57	0.217	0.612	0.645
Sl36	219	9	2.45	0.557	0.592	0.060
Sl65	194	15	2.35	0.196	0.574	0.659
Sl57	220	8	4.14	0.627	0.759	0.173
Sl18	217	8	3.26	0.452	0.693	0.349
Sl17	207	12	6.94	0.517	0.856	0.396
Sl60	198	12	4.39	0.379	0.772	0.509
Sl6	213	12	5.86	0.850	0.829	-0.025
Sl26	215	27	11.82	0.735	0.915	0.197
Sl67	216	9	4.49	0.620	0.777	0.202
Sl1	215	12	3.01	0.442	0.667	0.338
Sl47	215	6	2.07	0.447	0.517	0.136
Sl71	215	11	3.71	0.581	0.731	0.204
Mean	212 (2.2)	11.3 (1.5)	4.39 (0.74)	0.509 (0.051)	0.715 (0.033)	0.296 (0.059)
Mean (progeny)	101 (1.2)	7.5 (0.60)	3.93 (0.47)	0.426 (0.05)	0.698 (0.037)	0.386 (0.063)
Total	-	147	-	-	-	-

### Genetic differentiation and spatial structure

**Figure 3.**
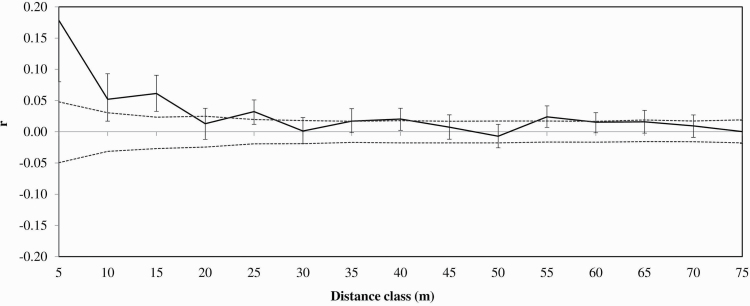
Genetic correlation coefficient (*r*) for increasing distances classes between *Styphelia longissima* individuals at 13 microsatellite loci. 95 % upper and lower confidence error bars determined from 999 bootstraps.

STRUCTURE analysis indicated little support for K > 1, and ΔK values were low for all values of K [see **Supporting Information Table S1** and **Figure S1**]. When considering all genotyped adult individuals in the population, there was no significant relationship between genetic and geographic distance (Mantel test; *P* = 0.061). Principal co-ordinate analysis of pairwise genetic distance revealed no distinct clustering. However, spatial auto correlation analysis revealed fine scale genetic structure, with a positive significant correlation between the genetic relatedness of individuals up to 20 m, which is the distance where *r* first changed from significant to non-significant (Fig. 3). This indicates a genetic patch size of less than 20 m, since beyond this distance, *r* was consistently non-significant, reflecting a stabilizing profile.

### Mating system and realized pollen dispersal

Estimated outcrossing rate (±SE) was considerably less than one (*t*_m_ = 0.66 (0.10)). Mating system estimates (±SE) were: parental inbreeding coefficient (*F*_m_) 0.27 (0.01); singlelocus outcrossing rate (*t*_s_) 0.30 (0.06); biparental inbreeding rate (*t*_m_-*t*_s_) 0.36 (0.07); correlation of paternity (*r*_p_) 0.62 (0.18); parental neighbourhood size (1/*r*_p_) 1.6. Mating system estimates from analysis of *only* large families were similar to estimates from analysis of the entire seedling cohort [see **Supporting Information Table S2**].

Of the 37 seedlings included in the paternity assignment, 26 were assigned at the 95 % confidence level, six at the 80 % confidence level and two were assigned the most likely sire. The remaining three progeny had negative LOD scores and were excluded from further analysis. A total of 13 progeny (38 %) were assigned identical maternal and paternal genotypes and contained no non-maternal alleles, and were therefore were classified as selfs. The ΔLOD scores were generally higher for assigned sires of seeds produced from selfing, indicating a higher degree of confidence when assigning sires for selfing events, compared with outcross events **[see**  **Supporting Information Figure S2****]**. The average exclusion probability across all loci was 99.92 %.

**Figure 4.**
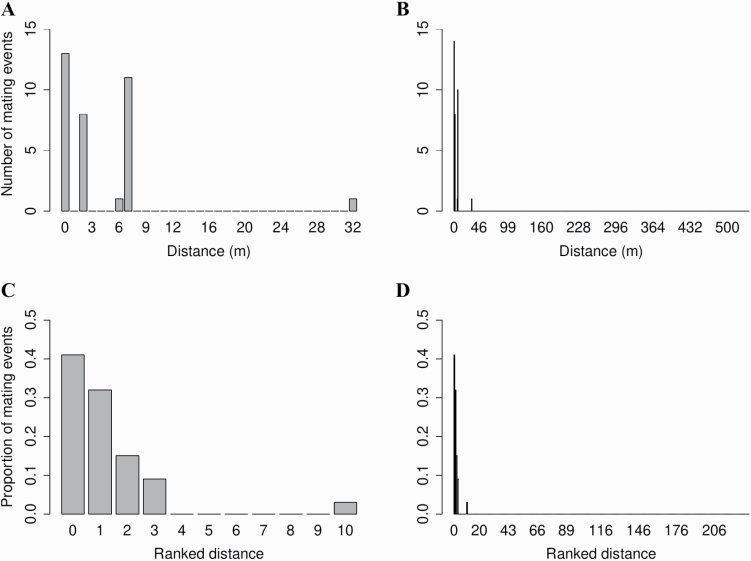
Number and proportion of mating events over distance and ranked distance between plant pairs of *Styphelia longissima*. Distance 0 represents a self. The X-axis shows (A) the maximum distance at which outcrossing occurred; (B) the maximum distance between two individuals; (C) the maximum distance rank at which outcrossing occurred; (D) the maximum distance rank between two individuals.

From paternity assignment, the observed outcrossing rate was 0.62, which was equivalent to the MLTR estimate (*t*_m_ = 0.66). Total number of sires ranged 1–4, while number of outcross sires ranged 0–3, for 3–15 seeds per family (Table 2). Mean realized pollen dispersal was 3.8 m (±0.4) when considering both selfing and outcrossing events, and 6.2 m (±1.4) when considering only outcrossing events. Short distance mating was common, with 95 % of outcrossing events occurring over a distance of 7 m or less (Fig. 4). The maximum distance between two mates was 32 m. The ranked distance distribution showed that 55 % of outcrossing events occurred between nearest-neighbours and 97 % of outcrossing events occurred between the three nearest-neighbours (Fig. 4). Both distributions demonstrated extreme leptokurtosis, with kurtosis values of 214 for the distance distribution and 145 for the ranked distributions.

**Table 2.** Number of total sires and outcross sires, outcrossing rate and mean pollen dispersal for seeds of *Styphelia longissima*. Values calculated from paternity analysis in CERVUS.

	*n*				
Maternal Plant ID	Seeds	Total sires	Outcross sires	Proportion outcrossed	Mean pollen dispersal (m)
1	3	1	0	0	0
2	12	2	1	0.67	1.33 (0.28)
3	15	4	3	0.60	5.87 (2.07)
4	4	2	2	1	6.75 (0.25)
Total	34	9	6	-	-
Mean	-	2.25 (0.63)	1.5 (0.65)	0.57	4.65 (0.39)
Overall mean	-	-	-	0.62	3.85 (0.42)

## Discussion

The maintenance of genetic diversity, both now and in the future, including strategies to manage genetic erosion, is an often overlooked yet key consideration for conserving critically endangered species and their adaptive potential (Laikre *et al*. 2020). An initial baseline characterization of genetic diversity, including the ecological genetic drivers influencing its spatial structure, is an essential first step towards implementing these strategies. In this study, the only known population of *S. longissima* was found to be characterized by surprisingly high levels of genetic diversity, with fine-spatial-scale genetic neighbourhoods of related individuals within which the vast majority of mating occurs. Assessment of mating patterns showed low paternal diversity and extensive inbreeding, in the form of selfing and biparental inbreeding; however, there was no indirect evidence of inbreeding depression in the progeny.

### *Styphelia longissima* displays surprisingly high levels of genetic diversity

Small isolated populations are predicted to display the effects of genetic drift, inbreeding depression, purging of genetic load and genetic bottlenecks coupled with founder effects, resulting in the erosion of genetic diversity (Karron 1991; Ellstrand and Elam 1993; Hamrick and Godt 1996; Gitzendanner and Soltis 2000; Crnokrak and Barrett 2002; Cole 2003; Frankham *et al*. 2017). In *S. longissima*, mean expected heterozygosity (*H*_E_ = 0.71) was higher than that reported in a review of microsatellite data for Australian shrubs (*H*_E_ = 0.60, *n* = 47) and plants with a localized distribution (*H*_E_ = 0.59, *n* = 31) (Broadhurst *et al*. 2017). In this review, a localized distribution was defined up to 100 km, which is much greater than the extreme endemism of *S. longissima* (0.21 km^2^). Therefore, measures of expected heterozygosity in cases of extreme endemism, like that of *S. longissima*, may not be fully represented. When compared with diversity measures from other microsatellite-based studies on more narrowly endemic Australian shrubs, including those with localized distributions restricted to banded ironstone formations in Western Australia (Byrne *et al*. 2019), both mean number of alleles per locus and mean expected heterozygosity were highest in *S. longissima*. Considering the extremely narrow distribution and small population size of *S. longissima*, overall levels of genetic variation were surprisingly high.

High levels of genetic variation in small and geographically isolated populations of rare Australian plants have previously been attributed to strong gene flow among populations (Maguire and Sedgley 1997), the selection for heterozygosity in small populations (i.e. the James Effect) (Coates 1988; James 2000; Rossetto *et al*. 1995), pollinators facilitating wide outcrossing and high paternal diversity (Hopper 2009; Krauss *et al*. 2017), strong inbreeding depression and delays in the reduction of genetic diversity following a natural reduction in population size or anthropogenic disturbance (Coates 1988; Rossetto *et al*. 1995). In the case of *S. longissima*, which is restricted to a single population with no opportunity for gene flow, one possible explanation is that the current levels of genetic diversity reflect pre-disturbance values and insufficient time has passed for a detectable reduction in genetic diversity. This is consistent with the OCBIL theory, which suggests that the retention of genetic diversity is a feature of plants in old, climatically buffered and infertile landscapes such as kwongan heathland (Hopper 2009). In support, *Leucopogon obtectus*, a close relative of *S. longissima* which exists in several small populations within kwongan heathland, was also found to have high levels of genetic diversity (Zawko *et al*. 2001).

### Inbreeding is a feature of *S. longissima*

Multiple lines of evidence indicate that inbreeding is a feature of *S. longissima*. These include: (i) a significant deficit of observed heterozygosity from HWE expectations at 12 of 13 loci; (ii) an overall fixation index of *F* = 0.30; (iii) equivalent fixation indices in the adult and progeny cohorts; (iv) high estimates of selfing (MLTR, *t*_*s*_ = 0.3 and CERVUS, 38 % of individuals assigned paternity as a self); (v) high estimate of bi-parental inbreeding (*t*_m_  *- t*_s_ = 0.36); (vi) high estimate of correlation of paternity (*r*_p_ = 0.62), (vii) restricted pollen dispersal (97 % of outcrossing events among three nearest-neighbours), and (viii) strong SGS. Despite this, levels of allelic diversity and expected heterozygosity were surprisingly high.

Technical artefacts, such as null alleles, could explain the observed deficit of heterozygotes, but this seems unlikely given the consistent heterozygote deficit across all but one locus. Here, a Wahlund effect appears a more likely biological explanation. The Wahlund effect describes a deficiency in observed heterozygosity compared with expected heterozygosity due to population substructure, where a departure from Hardy–Weinberg Equilibrium (HWE) occurs because subpopulations that have different allelic frequencies are considered as one population, even if the subpopulations are in HWE (Wahlund 1928). The fine-scale genetic structure, restricted pollen dispersal and small parental neighbourhood size found in *S. longissima* indicates the population consists of multiple small neighbourhoods, within which the vast majority of matings occur. Consequently, given the strong spatial genetic structuring at interplant distances less than 20 m, allelic frequencies are not equal across the population, and because of restricted pollen dispersal, realized mating is predominantly among relatives and departs significantly from random mating.

### Does inbreeding matter for conservation of *S. longissima*?

The non-viability of approximately 20 % of fruit and the low germination rate of seeds collected in this study could indeed be early signs of inbreeding depression. However, equivalent fixation indices between the adult and seedling cohorts suggests that inbreeding depression may be negligible in *S. longissima—*that is fitness declines are not associated with inbreeding and more homozygous inbred progeny are not being selected against as they germinate, establish and mature. This appears surprising, as inbreeding depression is a common feature of plants (Husband and Schemske 1996), and is a major concern for the conservation and recovery of threatened species (Hedrick and Kalinowski 2000). With inbreeding depression, high levels of genetic diversity can be maintained in a population despite prevalent inbreeding, through selection against inbred offspring (James 2000). When this occurs, levels of heterozygosity increase every generation and the fixation index decreases. This appears not to be the case for *S. longissima* and is an unlikely explanation for high heterozygosity in the standing population.

Inbreeding depression can be negligible in natural plant populations. For example, there was no association between early seedling growth and heterozygosity in seedlings of the anciently fragmented *Eucalyptus caesia*, suggesting an absence of inbreeding depression at least at this early life stage (Bezemer 2018; Bezemer *et al*. 2019). In *E. caesia*, a long history of population isolation and inbreeding appears to have purged the genetic load, a hypothesis supported by relatively low levels of heterozygosity. In natural populations of *Arabidopsis thaliana*, inbreeding depression was absent but heterosis with wide outcrossing was widespread and substantial, indicating an important role for drift shaping genetic variation (Oakley *et al*. 2019). Similarly, heterosis was found to increase ecological amplitude following wide outcrossing among plants from populations 20 km apart in *Banksia ilicifolia* (Heliyanto *et al*. 2006). Heterosis can also occur at fine spatial scales. For example, near-neighbour crosses showed higher fitness than nearest-neighbour crosses in *Anigozanthos manglesii*, consistent with the scale of SGS in this population (Ayre *et al*. 2019). This result reflects a common observation of an optimal outcrossing distance that extends beyond a genetic patch size within which mating results in relatively inbred offspring (Waser 1993). Further analyses are required to accurately assess the fitness consequences of inbreeding in *S. longissima*.

### Conservation implications of the mating system of *S. longissima*

*Styphelia longissima* has been shown here to be self-compatible and have a mixed mating system with substantial inbreeding through selfing and bi-parental inbreeding. Ultimately, what is unclear, yet of most significance for conservation, is whether these mating system parameters reflect pre-disturbance values, or are already reflecting disturbance impacts from habitat fragmentation and reduced population size. Are there already fewer pollinators (or even alien *Apis mellifera*) with altered behaviours impacting mating and eroding genetic diversity of progeny? Although the outcrossing rate (*t*_m_ = 0.66) for *S. longissima* is consistent with that reported for other rare insect pollinated shrubs in Western Australia (Yates and Ladd 2005; Coates *et al*. 2006), the widespread generality of temporal and spatial variability in outcrossing rate within and among populations and species globally (Whitehead *et al*. 2018) highlights the need for caution in interpreting mating system results for *S. longissima*. However, it is clear that ecological factors such as habitat disturbance and fragmentation can be a key a driver of this variability, by reducing pollinator abundance, changing visitation behaviour and reducing levels of outcrossing (Ayre *et al*. 1994; Sampson *et al*. 1996; Eckert *et al*. 2010).

While outcrossing rates were equivalent, the estimates of bi-parental inbreeding (*t*_m_ - *t*_s_ = 0.36) and correlated paternity (*r*_p_ = 0.62) were considerably greater than in many other insect pollinated species (Millar *et al*. 2000; Yates and Ladd 2005; Coates *et al*. 2006). These estimates were consistent with conclusions drawn from paternity assignment (97 % of outcrossing events among three nearest-neighbours), reflecting the majority of mating occurring between related mate pairs. This reflects low paternal diversity and is consistent with a very low parental neighbourhood size estimate of < 2 pollen donors (1/*r*_p_ = 1.6). Again, it is unclear to what extent this might also reflect pre-disturbance measures. However, increased paternal diversity has been shown to positively influence fitness in other species (Breed *et al*. 2012, 2014). An opportunity for *in situ* conservation is to experimentally assess whether there are fitness benefits for *S. longissima* from increasing paternal diversity within individual maternal plants. This could be achieved experimentally through manipulating pollinations with paternally diverse pollen mixes, and/or by manipulating plant density, whereby interplant movement by pollinators may be increased if plants are physically crowded. For this, a more precise understanding of pollinators and their movements is essential.

### Population size and demography

The number of individuals found during the current survey (947) was substantially less than the 2188 individuals reported in 2007 (Woodman Environmental Consulting Pty Ltd 2008), and reveals a severe decline in population size of 57 % over the past 11 years. The only survey prior to this was in 2003 when 300–400 individuals were found (Woodman Environmental Consulting Pty Ltd 2003), suggesting significant recruitment between 2003 and 2007. At the time of sampling (June 2018), 94 % of individuals were deemed healthy and in full flower, and 12 % were juvenile. In 2007, only 58 % of the plants were deemed healthy and <1 % were juvenile. Despite the large proportion of juveniles observed, mortality is clearly outweighing recruitment. The reasons for population decline over the past 11 years remain unclear but need to be addressed. The longevity of the soil-stored seed bank, as well as the role of fire on seed germination may be significant, as it is likely that recruitment occurs following, or between, fire events, as seen in other Ericaceae (Ooi *et al*. 2004,^ 2006^). Concurrently, on-going demographic monitoring is essential.

### Conservation recommendations

The biggest concern for the conservation of *S. longissima* is that only one natural population exists. In the case of a catastrophic event which eradicates the population, the species will become extinct in the wild. This concern is also pertinent for losses of genetic diversity, whereby the loss of alleles is irrecoverable because there are no other populations that can supplement these losses. Thus, an ongoing genetic monitoring plan should be implemented which can utilize the measures of genetic diversity established in this study as a baseline to identify loss of genetic diversity.

Currently, *S. longissima* is protected *ex situ* in tissue culture collections maintained by the Kings Park Botanic Gardens and in seed collections at the Threated Flora Seed Centre, Department of Biodiversity Conservation and Attractions in Western Australia. The genetic representation of these collections, however, is unknown. Due to the SGS identified in this study, *ex situ* collections should be genetically optimized by sampling material (seed or tissue culture) across the current distribution and ensuring that samples from within 20 m genetic neighbourhoods are minimized while samples from among neighbourhoods are maximized, thereby efficiently capturing representative genetic diversity.

Whether heterosis is associated with outcrossing between mates beyond the 20 m genetic neighbourhood, and whether heterosis is associated with increasing paternal diversity, needs to be confirmed. If it is, then increased seedling fitness with heterosis from wide outcrossing, and increased maternal fitness with increased paternal diversity, enable an active management plan for conservation benefits. These may be achieved by artificially dispersing seed and/or planting seedlings within the natural population to erode SGS. Spatial homogenization means that nearest-neighbour mating does not then equate to mating between relatives, which results in fitness benefits from heterosis.

Ultimately, translocation will be critical to reduce *in situ* extinction risk (Commander *et al*. 2018). While current *ex situ* collections provide a buffer against extinction (Harris 2013), *in situ* survival is precarious. Harris (2013) demonstrated that seedling production from micropropagated hypocotyl tissue is feasible and, therefore, the mass propagation of clonal cultures could be used in addition to seed in establishing future restorations trials. In such trials, there is an opportunity to address the issue of use of cuttings from wild plants vs natural seed vs seed that is the product of hand pollination for wide outcrossing, in a context of assessing strategies to best manage genetic diversity while achieving translocation objectives (Krauss *et al*. 2002). When establishing a new population, the spatial arrangement of genotypes should be randomized to minimize SGS. Furthermore, given the 20 m genetic neighbourhood size identified, approximately 430 individuals would be required to represent all the neighbourhoods present in the natural population. These recommendations are specific examples of a growing recognition of the benefits of incorporating genetic knowledge into optimizing the design and establishment of translocation populations (Bragg *et al*. 2020). In addition, understanding the ecophysiological and genetic capacity of plants in situ to adapt to rapid climatic change, as well as defining relevant thresholds, will enhance our understanding of population viability and evolutionary potential.

## Supporting Information

The following additional information is available in the online version of this article—

**Table S1**. STRUCTURE Harvester results when using the Evanno method to determine which K value best fit the data for K = 2–5.

**Figure S1**. Patterns of genetic structure generated in STRUCTURE across 13 microsatellite loci for 220 individuals of *Styphelia longissima*. Bar plots for K = 2–5 are shown.

**Table S2**. Mating system estimates (+SE) for *S. longissima* for only large families and all families. Estimates include parental inbreeding coefficient (F_*m*_), multilocus outcrossing rate (t_*m*_), singlelocus outcrossing rate (t_*s*_), bi-parental inbreeding rate (t_*m*_-t_*s*_), correlation of paternity (r_*p*_) and parental neighbourhood size (1/r_*p*_).

**Figure S2**. Relative confidence for the paternity assignments from CERVUS of seeds of *Styphelia longissima*, with realized pollen dispersal categories shown. LOD score is an estimate of the likelihood that a candidate parent is the true parent.

plab005_suppl_Supplementary_Table_Figures

plab005_suppl_Supplementary_Material
