# Supplementary material for: Optimising the conservation of genetic diversity of the last remaining population of a critically endangered shrub
Source: AoB Plants. 2021 Jan 9;13(1):plab005. doi: 10.1093/aobpla/plab005 (PMC7885199; doi:10.1093/aobpla/plab005)

Supporting Information

Table S1. STRUCTURE Harvester results when using the Evanno method to determine which K value best fit the data for K = 2 – 5.

K	Iterations	Mean LnP(K)	Stdev LnP(K)	Ln'(K)	|Ln''(K)|	ΔK
2	10	-9574.92	157.11	-133.69	268.73	1.33
3	10	-9401.88	67.07	173.04	90.30	1.15
4	10	-9299.14	123.74	102.74	67.34	0.86
5	10	-9244.74	114.19	54.40	59.61	0.74

Figure S1. Patterns of genetic structure generated in STRUCTURE across 13 microsatellite loci for 220 individuals of *Styphelia longissima*. Bar plots for K = 2 – 5 are shown.

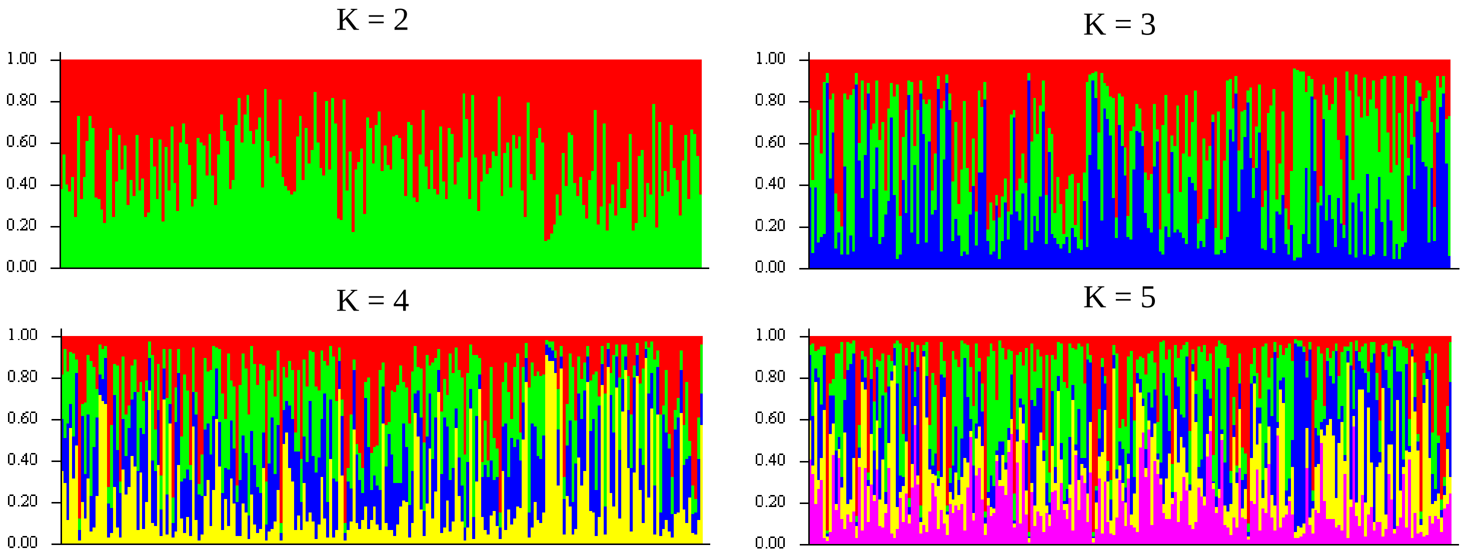

Table S2. Mating system estimates (±SE) for *S. longissima* for only large families and all families. Estimates include parental inbreeding coefficient (F_m), multilocus outcrossing rate (t_m), singlelocus outcrossing rate (t_s), bi-parental inbreeding rate (t_m-t_s), correlation of paternity (r_p) and parental neighbourhood size ($1/r_p$).

	F_m	t_m	t_s	t_m-t_s	r_p	$1/r_p$
Only large families	0.28 (0.01)	0.69 (0.07)	0.32 (0.11)	0.38 (0.08)	0.59 (0.14)	1.8
All families	0.27 (0.01)	0.66 (0.10)	0.30 (0.06)	0.36 (0.07)	0.62 (0.18)	1.6

Figure S2. Relative confidence for the paternity assignments from CERVUS of seeds of *Styphelia longissima*, with realized pollen dispersal categories shown. LOD score is an estimate of the likelihood that a candidate parent is the true parent.

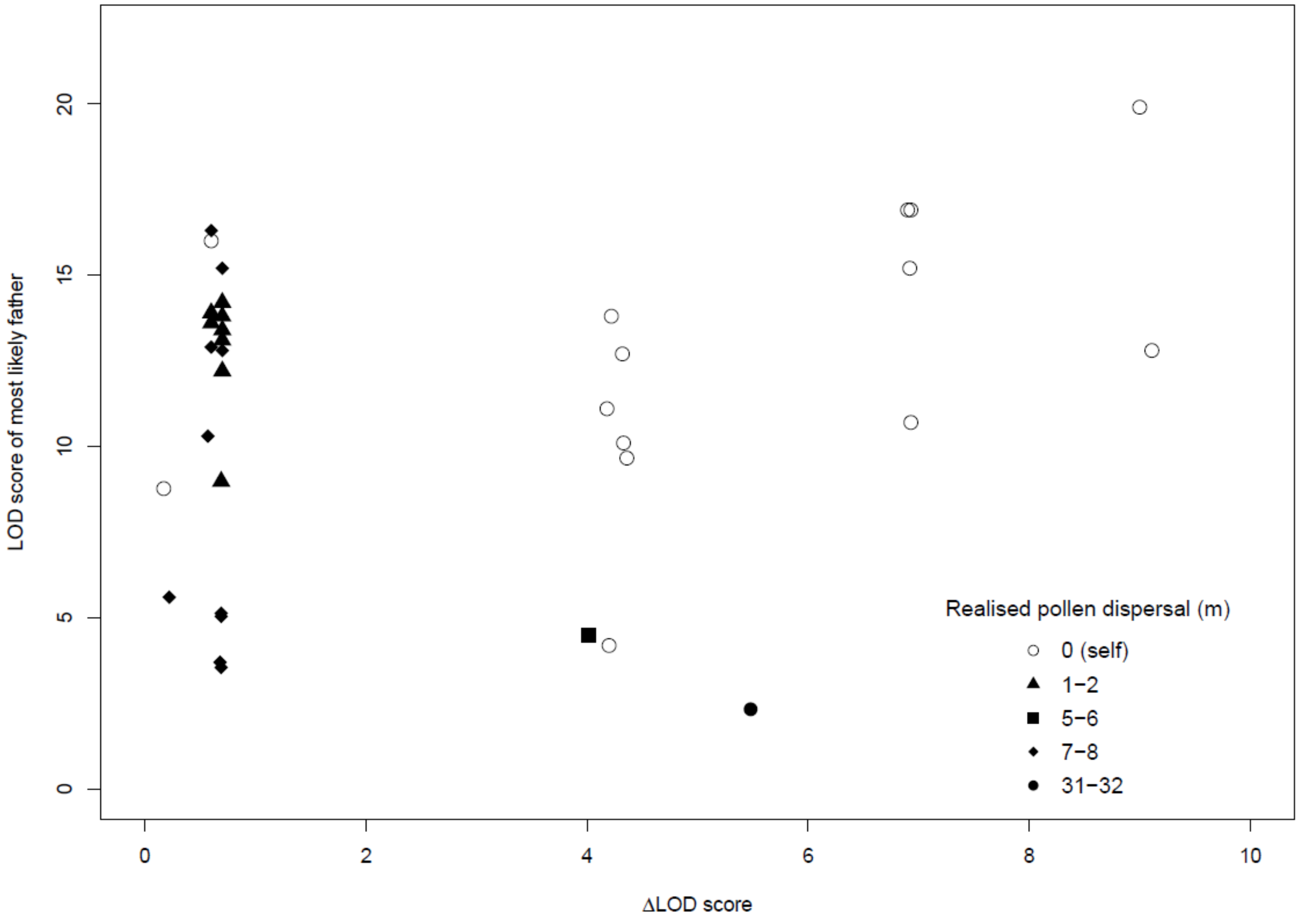

Supplement: plab005_suppl_Supplementary_Table_Figures [file plab005_suppl_supplementary_table_figures.pdf]
